# Editorial: Genetics of sudden unexplained death in children and young adults: state of the art, testing and implications for translational research, public health and forensic pathology

**DOI:** 10.3389/fmed.2023.1309179

**Published:** 2023-10-26

**Authors:** Simone Grassi, Vilma Pinchi, Oscar Campuzano, Antonio Oliva, Ramon Brugada

**Affiliations:** ^1^Department of Health Sciences, Section of Forensic Medical Sciences, University of Florence, Florence, Italy; ^2^Medical Science Department, School of Medicine, University of Girona, Girona, Spain; ^3^Cardiovascular Genetics Center, University of Girona-IDIBGI, Girona, Spain; ^4^Centro de Investigación Biomédica en Red Enfermedades Cardiovasculares (CIBERCV), Madrid, Spain; ^5^Department of Health Surveillance and Bioethics, Section of Legal Medicine, Fondazione Policlinico A. Gemelli IRCCS, Università Cattolica del Sacro Cuore, Rome, Italy

**Keywords:** sudden cardiac death, sudden death, forensic pathology, molecular autopsy, post-mortem genetic testing

Sudden cardiac death (SCD) still represents up to the 20% of all deaths worldwide, and is mainly caused by coronary artery disease, especially in people >40 years old (1). In contrast, the main cause of SCD in young population is represented by the so-called inherited arrhythmogenic syndromes (i.e., cardiomyopathies and channelopathies) (Martínez-Barrios et al.). Inherited arrhythmogenic syndromes share complex features like incomplete penetrance, variable expressivity and genetic overlap from a molecular point of view, and may present ambiguous patterns at macroscopic/microscopic evaluation (Martínez-Barrios et al.).

Thorough investigation of SCD cases is considered a public health priority (2), but the main limitation/challenge to the implementation of post-mortem genetic testing techniques (the so-called “molecular autopsy”) is still represented by the relatively low diagnostic yield (with high prevalence of variants of unknown significance).

Martínez-Barrios et al. reviewed the main aspects and evolutions of molecular autopsy, underlining a diagnostic yield up to 35% if adequate clinical information is collected and comprehensive genetic panels are chosen. In general, the authors stressed that many of the actual challenges of these techniques are given by issues that could be largely contained if good practices would be implemented at both local and global levels. Notably, the authors observed that up to 40% of samples obtained at autopsy do not meet the standards for molecular autopsy, a significant issue that is mainly due to poor sampling/storage and to the improper routine of fixing in formalin and embedding in paraffin solid samples regardless of their analytic use. Indeed, fresh tissues should be preferred when available because formalin-fixed, paraffin-embedded samples are exposed to a higher risk of low-quality and/or low-quantity DNA, due to DNA fragmentation, chemical crosslinking, deamination of cytosine bases and production of abasic sites (3). To contain these risks, when fresh tissues are not available, the use of DNA extraction methods specific for formalin-fixed, paraffin-embedded samples is highly recommended (4). Finally, in order to overcome the issue represented by high rate of variants of unknown significance and failure to correctly interpret genetic results, the authors suggested the production of forensic-targeted guidelines and the creation of national multidisciplinary referral units (Martínez-Barrios et al.).

An example of the potential reached by rigorous analysis of cases affected by an inherited arrhythmogenic syndrome has been reported by Greiner et al., who investigated a multigenerational family affected by Brugada Syndrome—BrS—, one of most prevalent inherited arrhythmogenic syndromes. Currently, the only gene with a strong association to BrS is the *SCN5A* gene, being responsible for 25% of diagnosed cases (5). Their group identified a linkage region on chromosome 3 that did not contain *SCN10A* or *SCN5A* genes. In the found region, a variant (p.A280V) was reported in the *GPD1L* gene, a minor gene associated with BrS (5). As reported by the authors, the clinical interpretation of this variant had previously been hindered by factors like the significant dimension of the linkage region and its relatively high allele frequency. Therefore, they opted for an articulate approach combining high-depth whole exome sequencing for the proband, SNP-based linkage analysis of affected cases, and sequencing data of SNPs associated with risk of BrS, allowing the identification of the found rare variant as pathogenic in the analyzed family.

Failing to make diagnosis in carriers of pathogenic variants entails severe ethical and medico-legal implications. As reported by Brlek et al., relatives of cases of SCD due to inherited arrhythmogenic syndromes do have a right to choose to consider genetic testing and, in case, opt for preventive interventions even in the absence of symptoms. However, discovering the genetic underpinnings of inherited arrhythmogenic syndromes does not mean only to make more and earlier diagnoses but also to better understand the pathogenic mechanisms of these disorders. At this regard, Wojcik et al. focused on sudden unexpected pediatric deaths, stressing that up to date only nonspecific autopsy features are reported in these cases, with the pathophysiology of this condition still largely unclear. Hence, the authors proposed a novel phenotyping strategy valid for cases younger than 10 years. They advised against the a priori hypothesis of a relationship between extrinsic, intrinsic, and developmental risks, suggesting that sudden unexplained deaths in pediatrics should be treated “*as a broad phenotype of undiagnosed disease conforming to a distinct pattern in human survival*.”

In conclusion, awareness regarding the importance of molecular autopsy in SCD is increasing together with rigorous and promising evidence about its causes and its diagnosis, but accurate and targeted autopsy protocols, stringent criteria for analysis, classification and (re)interpretation of genetic variants, and concentration of analyses in referral centers remain the main tools to challenge the status quo (6–8).

## Author contributions

SG: Writing – original draft, Writing – review & editing. VP: Writing – original draft, Writing – review & editing. OC: Writing – original draft, Writing – review & editing. AO: Writing – original draft, Writing – review & editing. RB: Writing – original draft, Writing – review & editing.
